# ERBB3 knockdown induces cell cycle arrest and activation of Bak and Bax-dependent apoptosis in colon cancer cells

**DOI:** 10.18632/oncotarget.2094

**Published:** 2014-06-11

**Authors:** Hyunji Lee, Hyunjung Lee, Hyunjung Chin, Kyoungmi Kim, Daekee Lee

**Affiliations:** ^1^ Department of Life Science Ewha Womans University, Seoul, S. Korea; ^2^ GT5 program, Ewha Womans University, Seoul, S. Korea

**Keywords:** ERBB3 targeting, Cell cycle arrest, Apoptosis, Bak, Bax

## Abstract

ERBB3 is an emerging target for cancer therapy among the EGFR family. Contrary to resistance against EGFR and ERBB2 targeting, the genetic inhibition of ERBB3 results in anti-tumorigenic in HCT116 colon cancer cells harboring constitutively active KRAS and PIK3CA mutations. Still, the anti-tumorigenic molecular mechanism has not been defined. We demonstrated in this study that ERBB3 knockdown resulted in cell cycle arrest and activation of Bak and Bax-dependent apoptosis. Apoptosis was irrelevant to the majority of BH3-only pro-apoptotic proteins and correlated with the transcriptional upregulation of Bak and p53-dependent Bax translocation. Treatment with LY294002, a PI3K inhibitor, resulted in cell cycle arrest without apoptosis and a concomitant down-regulation of cap-dependent translation by the suppression of the PI3K/AKT/mTOR pathway. However, the inhibition of cap-dependent translation by ERBB3 knockdown occurred without altering the PI3K/AKT/mTOR pathway. In addition, ERBB3 knockdown-induced cell cycle arrest was observed in most colon cancer cells but was accompanied by apoptosis in p53 wild-type cells. These results indicate that ERBB3 is a potential target for EGFR- and ERBB2-resistant colon cancer therapy.

## INTRODUCTION

The ERBB family includes ERBB1 (known as EGFR), ERBB2, ERBB3 and ERBB4, a single transmembrane receptor tyrosine kinase (RTK) that forms homo- or heterodimers with other members, phosphorylating the cytoplasmic tail of the receptor to recruit the adaptors for downstream signaling pathways [1]. Because the overexpression or hyperactive mutations in ERBB family drive uncontrolled cell proliferation and survival, several drugs targeting the ERBB family have been used to treat cancer patients [2]. Preclinical studies with small molecule tyrosine kinase inhibitors (TKIs) and monoclonal antibodies (mAbs) targeting EGFR or ERBB2 have indicated that these anti-tumorigenic actions partly result from the elevated level of apoptosis. For example, treatment with gefitinib or erlotinib, a TKI for EGFR, causes enhanced apoptosis in several cancer cells in vitro [3-5] or in xenograft tumor growths from breast cancer patients [6] and EGFR-dependent non-small cell lung cancer (NSCLC) cells [7], respectively. The mAb targeting of either EGFR [8] or ERBB2 [9] also generates an increased rate of apoptosis in cancer cells in vitro.

In contrast to EGFR and ERBB2, the potency of ERBB3 as a therapeutic target in cancer has only recently been recognized. ERBB3 is known to have impaired RTK activity, unlike other ERBB receptors [10]. However, ERBB3 is the preferred partner of ERBB2, and ERBB2/ERBB3 heterodimers induce the most potent downstream signals for cell proliferation [11, 12]. The most intriguing feature of ERBB3 is that it contains multiple binding sites for the p85 regulatory subunit of PI3K [13, 14], allowing it to activate the AKT pathway, a critical signal in a variety of cancers [15]. Not surprisingly, the overexpression of ERBB3 has been frequently detected in many types of human cancers [16], and accumulating evidence strongly suggests that ERBB3 plays a crucial role in cancers driven by EGFR and ERBB2 [17]. Moreover, the activation of the ERBB3-mediated PI3K/AKT pathway renders cancer cells sensitive or resistant to ERBB inhibitors, depending on the cellular context [18, 19]. Recent findings on trans-autophosphorylation ERBB3 activity [20] and oncogenic ERBB3 mutation in human cancers [21] have marked ERBB3 as an even more potent target for cancer therapy. The genetic and pharmacological inhibition of ERBB3 results in anti-tumorigenic activity in cancer cells, both in vitro and as xenograft [5, 22-27], and sensitizes lung cancer cells [27] and breast cancer cells [28] to lapatinib, a dual TKI of EGFR and ERBB2. An ERBB3 blockade results in the retardation of cell proliferation and elevated apoptosis in colon cancer cells [25, 29], however, the mechanism leading to apoptosis is unknown.

The Bcl-2-regulated intrinsic death pathway consists of anti-apoptotic, pro-apoptotic Bcl-2 family and Bcl-2-homology domain 3 (BH3)-only proteins. BH3-only proteins play a key role in mediating distinct apoptotic signaling, including signals elicited by targeting oncogenic pathways [30]. Apoptosis resulting from the inhibition of EGFR is known to be mediated by the activation of Bad in the mammary epithelial cells [31], by the induction of Bim in NSCLC cells [32-34] or by the induction of Puma in head and neck cancer cells [35]. ERBB2-targeted mAb induces apoptosis by reducing Bcl-2 or Mcl-1 expression in ERBB2-amplified breast cancer cells [36, 37]. Recently, it has been shown that lapatinib-induced apoptosis requires Bim and Puma in ERBB2-amplified breast cancer cells [38]. HCT116 cells express high levels of EGFR, ERBB2 and ERBB3, but barely detectable ERBB4 [29]. The targeting of either EGFR [39, 40] or ERBB2 [41] unveils a resistance that is most likely due to the *KRAS* and *PIK3CA* mutations harbored in wild-type HCT116 cells, activating the MAPK and AKT pathways constitutively required for efficient cell growth [42-44]. However, ERBB3 knockdown-induced apoptosis in HCT116 cells suggests that an alternative pathway led to the stimulation of apoptosis. In the present study, we have analyzed the molecular mechanisms related to the anti-tumorigenic effects of the ERBB3 knockdown in colon cancer cells. The ERBB3 knockdown in HCT116 cells results in apoptosis, mediated by the induction of Bak and the translocation of Bax. Moreover, cell cycle arrest occurs in most colon cancer cells and is accompanied by apoptosis in a number of cell lines, supporting the potential for ERBB3 as a target in colon cancer therapy.

## RESULTS

### ERBB3 knockdown results in G1 arrest and apoptosis in HCT116 cells

**Figure 1:**
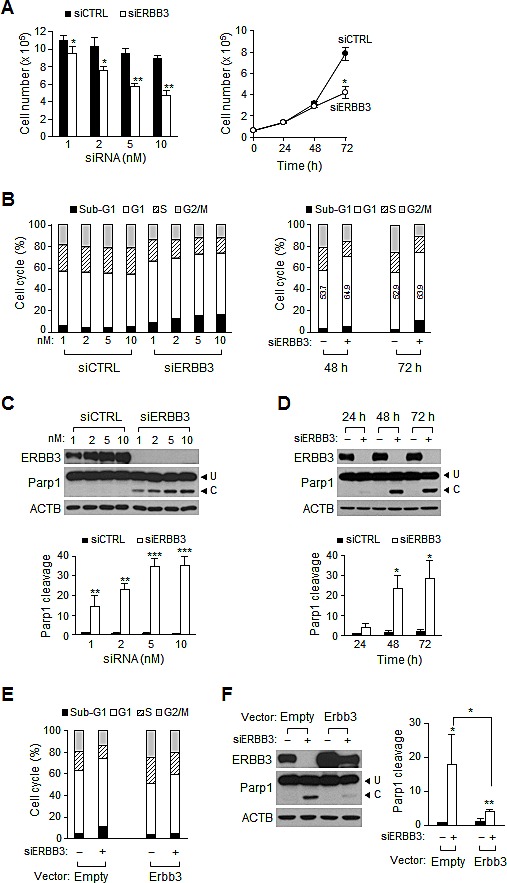
Effect of ERBB3 knockdown on cell proliferation, cell cycle and apoptosis in HCT116 cells (A) Viable cells were counted 72 h after treatment with different concentration of siRNA (left) or at different time points after treatment with 5 nM siRNA (right). (B) Cell cycle distribution was analyzed with FACS 72 h after transfection with different concentration of siRNA (left) or at different time points after treatment with 5 nM of siRNA (right). Numbers in open box indicate a percent of G1 populations. (C) Western blotting was performed using equal amounts of protein extracts prepared 72 h after transfection with different concentration of siRNA (top). The apoptotic index (Parp1 cleavage) was determined by the ratio of cleaved (C) to uncleaved Parp1 (U) (bottom). (D) The time course induction of Parp1 cleavage was determined after the treatment with 5 nM of siRNA using western blotting (top) and quantified (bottom). (E) Cells were analyzed with FACS or (F) western blotting (left) and Parp1 cleavage (right) was quantified after cells were transiently transfected with Erbb3 cDNA (Erbb3) expression vector or vector only (Empty), followed by siRNA treatment for 48 h. In B, D, E and F, – denotes treatment with siCTRL and +, with siERBB3. siERBB3 group was statistically compared to siCTRL group at each point, unless otherwise indicated.

Similar to anti-proliferation by individual siERBB3 [29], treatment with pooled siERBB3 also resulted in a decreased number of HCT116 cells in a dose-dependent manner (Figure 1A). Although ERBB3 proteins rapidly disappeared within 24 h (Figure 5B) after treatment, an inhibition of cell proliferation was manifested 72 h (Figure 1A). Cell cycle analysis revealed that siERBB3 caused an increase in the number of cells in sub-G1 and G1, indicating the occurrence of cell death and G1 arrest. G1-arrested cells had already accumulated 48 h (Figure 1B). Although treatment with 1 nM of siERBB3 was sufficient to deplete the ERBB3 protein near completely, the apoptosis measured by the proteolytic cleavage of Parp1 continued to increase, even at 5 nM of siERBB3 (Figure 1C), consistent with the sub-G1 fraction. Apoptosis sharply increased 48 h after siERBB3 treatment (Figure 1D). To determine whether the siERBB3-induced G1 arrest and apoptosis were due to the ERBB3 depletion, the cells were transfected with mouse *Erbb3* cDNA expression vector before knockdown. Overexpression of the cDNA maintained the basal level of ERBB3, even during the ERBB3 knockdown (Figure 1F). Cells transfected with cDNA showed an attenuation of the siERBB3-induced G1 arrest (Figure 1E) and apoptosis (Figure 1F), compared to cells with empty vectors, suggesting that G1 arrest and apoptosis is mediated by ERBB3 knockdown but not by off-target effects.

### ERBB3 knockdown alters the amount of pro- and anti-apoptotic proteins and Bax translocation

**Figure 2:**
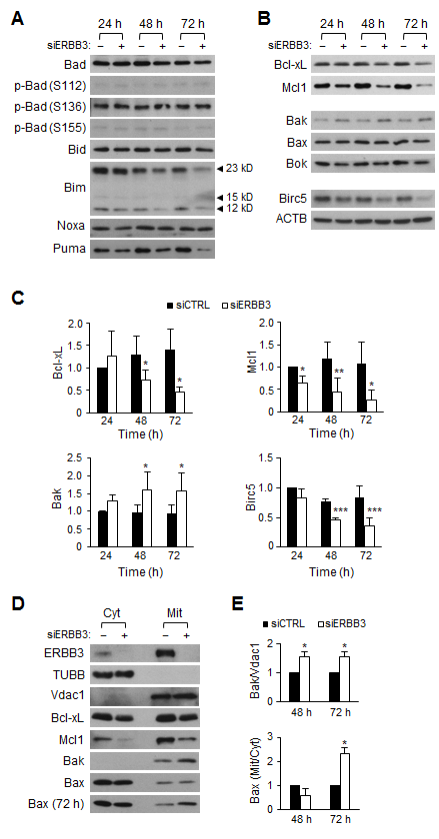
The time course changes in Bcl-2 family proteins levels after ERBB3 knockdown in HCT116 cells Western blotting was performed using protein extracts prepared daily after the treatment with 5 nM of siCTRL (–) or siERBB3 (+). Shown are representative (A) BH3-only proteins and (B) anti- or pro-apoptotic proteins, respectively. (C) The relative intensity of proteins in B compared to ACTB was normalized to that of siCTRL at 24 h. (D) Western blotting was performed using cytosol (Cyt) or membrane/organelle (Mit) fractions prepared 48 h after siRNA (5 nM) transfection. Bax (72 h) indicates blot using fractions prepared 72 h after transfection. TUBB and Vdac1 antibodies were used for the control of the Cyt and Mit fractions, respectively. (E) The relative band intensities of the mitochondrial Bak to Vdac1 (top), as well as the mitochondrial Bax to the cytosolic Bax (bottom), were normalized to the siCTRL group at each time point. siERBB3 group was statistically compared to siCTRL group at each point.

Considering the hyperactive KRAS and PIK3CA mutations in HCT116 cells, ERBB3 knockdown may not affect the amount of Bim or phospho-Bad. As expected, the ERBB3 knockdown did not alter the level of phospho-Bad (Figure 2A) and rather down-regulated the levels of Bad, Bid, Bim, Noxa and Puma (Figure 2A), indicating that BH3-only groups do not mediate apoptosis. Interestingly, the ERBB3 knockdown decreased the amount of Bcl-xL and Mcl1, an anti-apoptotic Bcl-2 family (Figure 2B,). However, the exogenous expression of Bcl-xL or Mcl1 alone did not block ERBB3 knockdown-induced apoptosis (Supplemental Figure 1). Among the pro-apoptotic Bcl-2 family, the amount of Bak only increased inversely to the levels of Bcl-xL and Mcl1 by ERBB3 knockdown (Figure 2B,). The amount of Birc5, a member of the inhibitors of apoptosis (IAP) family, was decreased by ERBB3 knockdown. To determine whether the changes in the protein levels were due to differential gene expression, the amount of transcripts was measured. In contrast to the consistent *BCL2L1* and *MCL1* mRNA levels, ERBB3 knockdown resulted in the upregulation of *BAK1* and downregulation of *BIRC5* transcripts levels (Supplemental Figure 2). These data suggest that changes in the amount of Bak and Birc5 proteins are primarily regulated at the transcriptional level, while those of Bcl-xL and Mcl1 proteins are regulated at the translational level.

Because translocations of the Bcl-2 family from the cytosol to the mitochondria play an important role in apoptosis [45], we compared the amount of proteins in the cytosol versus the mitochondria. The ratios of cytosol to membrane/organelle fractions were similar in Bcl-xL and Mcl1 (Figure 2D). As Bak proteins exist solely in the membrane fraction, the elevation of Bak was noted directly in the membrane/organelle fraction (Figure 2D,). Interestingly, no Bax translocation occurred within 48 h, although such an event manifested 72 h (Figure 2D, E), indicating that the mitochondrial Bax translocation is also involved in apoptosis.

### Bak and Bax mediate ERBB3 knockdown-induced apoptosis

**Figure 3:**
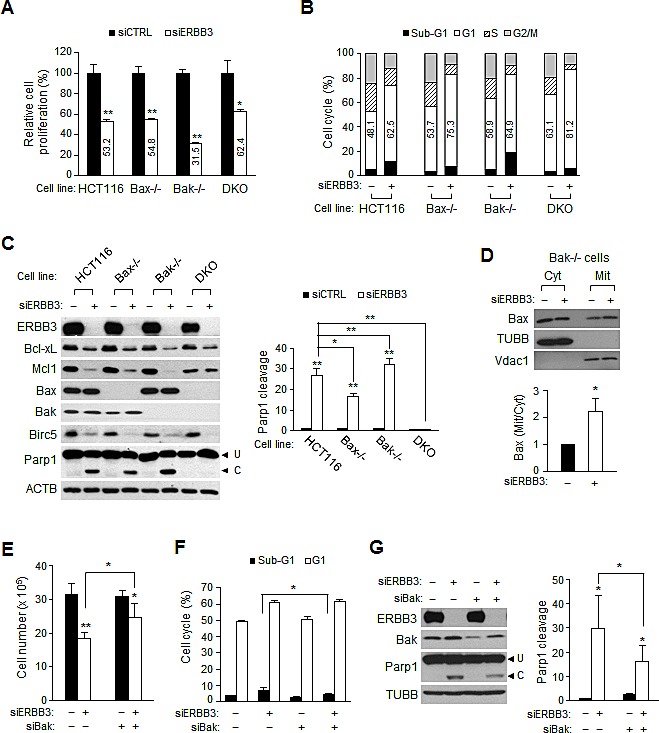
Bak and Bax-dependent apoptosis by ERBB3 knockdown in HCT116 cells (A) Cell proliferation and (B) cell cycle distribution relative to siCTRL (–) were determined 72 h after treatment with siERBB3 (+) in HCT116 mutant cells. Numbers in open box indicate a percent of G1 populations. (C) Protein extracts were prepared 72 h after siERBB3 treatment and analyzed by western blotting with indicated antibodies (left). Parp1 cleavage was quantified (right). (D) Protein extracts in the cytosol or membrane/organelle fraction 48 h after siERBB3 treatment were analyzed by western blotting in Bak-/- cells (top). The relative band intensities of mitochondrial Bax to cytosolic Bax were normalized to that of siCTRL (bottom). (E) Cell proliferation, (F) FACS and (G) western blotting (left) and quantification of parp1 cleavage (right) were performed after serial treatments with siBak and siERBB3. The cells were analyzed 48 h after the second siRNA treatment. For the knockdown, 5 nM of siERBB3 or 0.2 nM of siBak were used. siERBB3 group was statistically compared to siCTRL group at each point, unless otherwise indicated.

ERBB3 knockdown-induced G1 arrest and apoptosis were further analyzed in isogenic mutant HCT116 cells to investigate the significance of Bak and Bax. ERBB3 knockdowns resulted in the attenuation of cell proliferation in all three mutant cells, as in HCT116 cells (Figure 3A). However, the ERBB3 knockdown caused a slightly more reduced cell proliferation in Bak^−/−^ cells and an even more minor reduction in Bax^−/−^Bak^−/−^ DKO cells (Wild-type, 53%; Bax^−/−^, 55%; Bak^−/−^, 32%; DKO, 62% compared to siCTRL-treated group, respectively). FACS analysis displayed that the differential inhibition of cell proliferation in each cell line might reflect changes in the cell cycle distribution, compared to wild-type HCT116 cells (Figure 3B). In Bax^−/−^ cells, the ERBB3 knockdown resulted in less sub-G1 but greatly increased G1 populations; these changes were more dramatic in Bax^−/−^Bak^−/−^ DKO cells. In contrast, ERBB3 knockdown led to a larger sub-G1 population in Bak^−/−^ cells. The ratios of sub-G1 cells were correlated with the relative levels of Parp1 cleavage in each cell line (Figure 3C). The ERBB3 knockdown-induced downregulation of Bcl-xL and Mcl1 was independent of Bax or Bak. The ERBB3 knockdown caused a little less Parp1 cleavage in Bax^−/−^ cells and even more Parp1 cleavage in Bak^−/−^ cells, compared to wild-type HCT116 cells. This elevated apoptosis in Bak^−/−^ cells was likely due to the early Bax translocation, which occurred at 48 h (Figure 3D). To confirm the significance of Bak upregulation in apoptosis, wild-type HCT116 cells were pretreated with siBak before siERBB3 treatment to reduce the Bak level. Compared to those of ERBB3 knockdown alone, knockdowns of Bak and ERBB3 together resulted in a lessened inhibition of cell proliferation (Figure 3E), a smaller sub-G1 increase (Figure 3F), and less Parp1 cleavage (Figure 3G), respectively. These results further support the conclusion that Bak upregulation plays a partial role in ERBB3 knockdown-induced apoptosis.

### p53 mediates Bax translocation but does not affect G1 arrest

**Figure 4:**
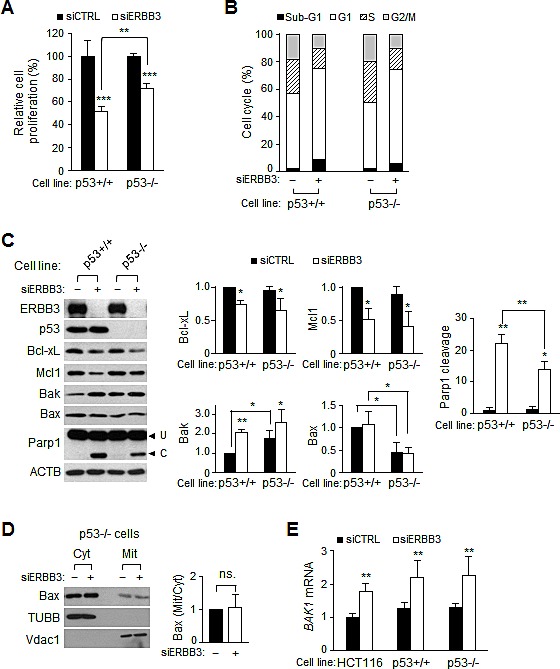
Effect of ERBB3 knockdown on cell proliferation, cell cycles and apoptosis in HCT116-p53 cells Cells were treated with 5 nM of siCTRL (-) or siERBB3 (+) for 72 h in A, B and D, for 48 h in C and for 24 h in E, respectively. (A) Viable cells were counted after siRNA treatment in p53^−/−^ (p53-/-) or parental p53^+/+^ HCT116 cells (p53+/+). (B) Cell cycle distribution was analyzed with FACS after the siRNA treatment in p53+/+ or p53-/- cells. (C) Equal amounts of protein extracts were analyzed with western blotting and the relative intensity of proteins compared to ACTB was normalized to that of siCTRL. Parp1 cleavage was also quantified. (D) Protein extracts in the cytosol or membrane/organelle fraction were analyzed by western blotting in p53-/- cells (left). The relative band intensities of mitochondrial Bax to cytosolic Bax were normalized to those of siCTRL (right). ns., statistically non-significant. (E) The relative amounts of *BAK1* mRNA were analyzed by qRT-PCR analysis using purified RNA from wild-type, p53+/+, and p53-/- cells. siERBB3 group was statistically compared to siCTRL group at each point, unless otherwise indicated.

p53 plays a critical role as a tumor suppressor, inducing cell cycle arrest and apoptosis. Therefore, we sought to determine whether p53 has influence on ERBB3 knockdown-induced G1 arrest and apoptosis using mutant p53^−/−^ cells. The ERBB3 knockdown-induced anti-proliferation, sub-G1 increase and Parp1 cleavage were all reduced partially in the p53^−/−^ cells, compared to the parental p53^+/+^ cells (Figure 4A,). However, the increase in the G1 population was similar between the two cell lines (Figure 4B), indicating that p53 is not involved with G1 arrest. Western blots (Figure 4C) showed that the reductions of Bcl-xL, Mcl1 and Birc5 occurred similarly in p53^−/−^ cells. The amount of Bax, one of the transcriptional targets of p53 [46], was reduced to a half level in p53^−/−^ cells but was not altered in the membrane/organelle fraction (Figure 4D). The ERBB3 knockdown-induced Bak increase still occurred in p53^−/−^ cells to a lesser extent because of the higher basal Bak level (Figure 4C). However, the basal and knockdown-induced *BAK1* mRNA levels were all similar in wild-type, p53^+/+^ and p53^−/−^ cells (Figure 4E), indicating a p53-independent *BAK1* mRNA expression. All together, these results reveal that p53 is not involved in G1 arrest, but the p53-dependent Bax translocation to the mitochondria participates in apoptosis driven by ERBB3 knockdown.

### ERBB3 knockdown attenuates cap-dependent translational pathway independent of PI3K/AKT/mTOR pathway

**Figure 5:**
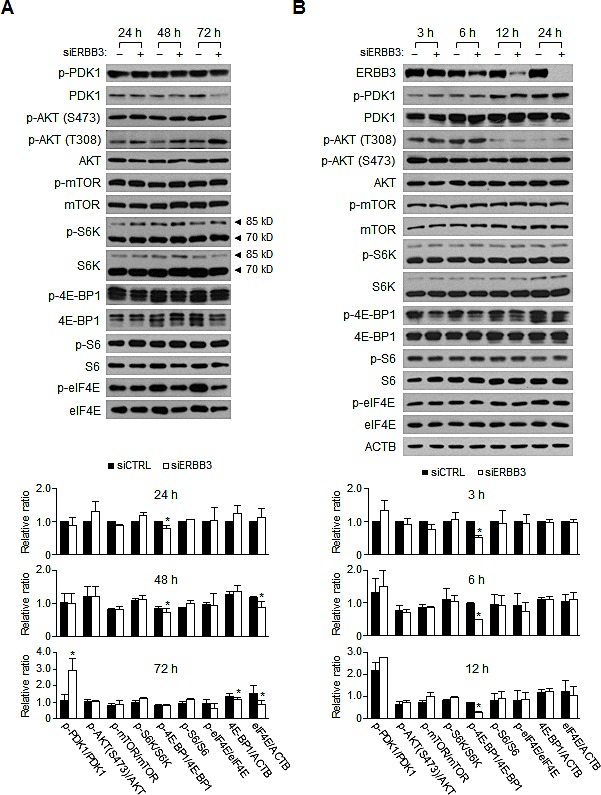
Changes in the signaling pathways induced by ERBB3 knockdowns in HCT116 cells (A) Western blotting was performed using the protein extracts prepared (A) daily or (B) at indicated hours after siRNA (5 nM) transfection. siERBB3 group was statistically compared to siCTRL group at each point. The relative intensity of proteins in A, B was normalized to that of siCTRL at 24 h (A, bottom) or that of siCTRL at 3 h (B, bottom). Only statistically significant differences are marked.

The PI3K/AKT/mTOR pathway activates the cap-dependent mRNA translation initiation by phosphorylating the eIF4E-binding proteins (4E-BPs) [47]. Because the activation of ERBB receptors precedes PI3K, it is likely that the mutant PI3K still stimulates the downstream AKT pathway, despite the ERBB3 knockdown. However, the ERBB3 knockdown still reduces the levels of the Bcl-xL, Mcl1 and Birc5 (Figure 2B,) as well as Cyclin D1 proteins (Supplemental Figure 4A, B), which are sensitive to eIF4E availability [48]. To understand this discrepancy, we analyzed ERBB3 knockdown-induced signaling changes by western blotting. The relative levels of phospho-PDK1, phospho-AKT and phospho-mTOR were similar between the siERBB3 and siCTRL-treated groups (Figure 5A), indicating that PI3K/AKT/mTOR is not altered by the ERBB3 knockdown. Interestingly, the ERBB3 knockdown decreased the level of phospho-4E-BP1 slightly, in contrast to the consistent levels of phospho-S6K and phospho-S6 (Figure 5A). Moreover, decreases in the level of phospho-4E-BP1 occurred rapidly without any significant changes in phospho-mTOR (Figure 5B), suggesting the presence of an mTOR-independent regulation of 4E-BP1 phosphorylation by ERBB3 knockdown. The phosphorylation of eIF4E was steady until 24 h by ERBB3 knockdown, but the phospho-eIF4E decreased thereafter, accompanied by reduced protein levels (Figure 5A,). The ERBB3 knockdown resulted in a downregulation of the phospho-EGFR, but the general phospho-MAPKs were relatively unchanged, except for phospho-p38 (Supplemental Figure 3A). Interestingly, the amounts of ERBB2, MAPK1/3, and p38 protein were decreased by ERBB3 knockdown (Supplemental Figure 3A), suggesting a broad attenuation of translation by the ERBB3 knockdown. Overall, these results suggest that the ERBB3 knockdown results in the reduction of phospho-4E-BP1 and eIF4E involved in cap-dependent translation without any significant alteration of the PI3K/AKT/mTOR pathway.

### LY294002 decreases cell proliferation by inducing cell cycle arrest

**Figure 6:**
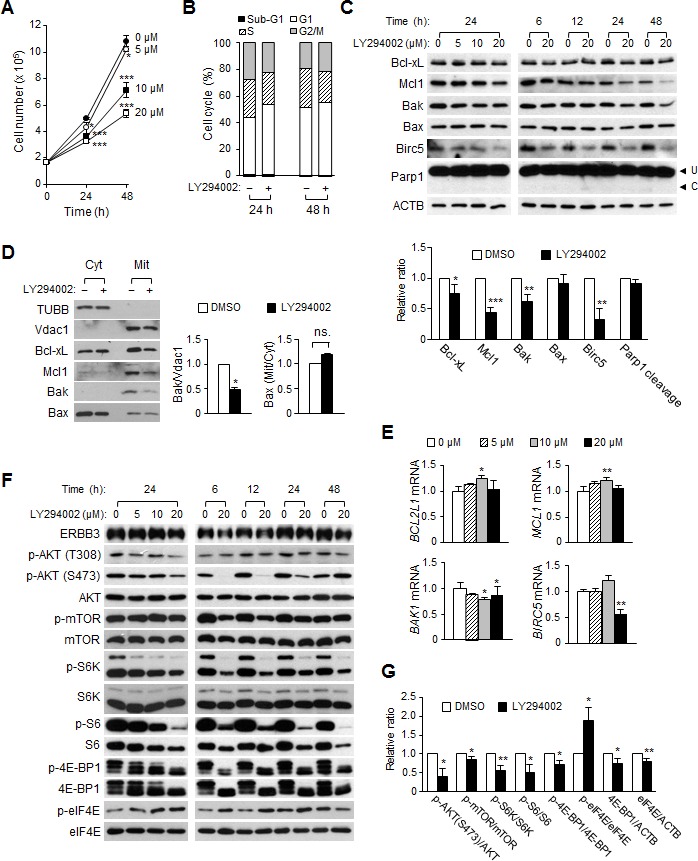
The effect of LY294002 on cell proliferation, cell cycles and apoptosis in HCT116 cells (A) Viable cells were counted daily after treatment with a different concentration of LY294002. (B) Cell cycle distribution was analyzed by FACS 24 and 48 h after treatment with 20 μM of LY294002 (+) or DMSO (–), respectively. (C) The amounts of anti- or pro-apoptotic proteins were analyzed by western blotting. Protein extracts were prepared either 24 h after treatment with different concentrations of LY294002 (left) or at different time points after treatment with 20 μM of LY294002 (right). The relative intensity of proteins (24 h with 20 μM of LY294002) compared to ACTB was normalized to that of DMSO-treated group and Parp1 cleavage was also quantified (bottom). (D) Western blotting was performed using cytosol or membrane/organelle fractions that had been prepared 24 h after 20 μM of LY294002 (+) or DMSO (–) treatment. The relative band intensities of the mitochondrial Bak to Vdac1 and of mitochondrial Bax to cytosolic Bax were normalized to that of the DMSO-treated group. (E) The relative amounts of mRNA were analyzed by qRT-PCR analysis using purified RNA from cells 24 h after the LY294002 treatment. (F) Shown are the representative western blots of the PI3K downstream pathway after LY294002 treatment. (G) The relative intensity of proteins (24 h with 20 μM of LY294002) in F was normalized to that of DMSO-treated group.

We analyzed the changes in signaling pathways and the levels of apoptotic proteins by treatment with LY294002, an inhibitor of PI3K, and compared the data to those by ERBB3 knockdown to identify ERBB3 knockdown-specific signaling pathways. LY294002 attenuated the proliferation of HCT116 cells in a dose-dependent manner (Figure 6A). FACS analysis revealed that LY294002 caused an increase in the G1 populations, indicating that a G1 arrest occurred (Figure 6B). In contrast to the ERBB3 knockdown, there was no sub-G1 increase (Figure 6B) or Parp1 cleavage (Figure 6C) by LY294002, indicating that the attenuation of cell proliferation was due to cell cycle arrest. The amounts of Bcl-xL, Mcl1, Birc5, Cyclin D1 and Cyclin E1 proteins decreased by LY294002 (Figure 6C and Supplemental Figure 4C,) similarly as in the ERBB3 knockdown. On the other hand, neither an increase in the Bak proteins (Figure 6C) nor a Bax translocation (Figure 6D) occurred by LY294002. The amount of Bak, instead, decreased by LY294002 (Figure 6C,) along with a slight reduction in the *BAK1* mRNA level (Figure 6E). The levels of *BCL2L1* and *MCL1* mRNA were relatively consistent (Figure 6E), suggesting that the reduction of Bcl-xL and Mcl1 are regulated at the translational level, similar to the siERBB3 treatment. The downregulation of *BIRC5* mRNA (Figure 6E) was also similar to siERBB3 treatment.

Analysis of downstream signaling changes by LY294002 showed that the reduction of phospho-AKT and phospho-mTOR levels occurred rapidly after LY294002 treatment (Figure 6F,), although those levels had recovered at 48 h after treatment. LY294002 attenuated the phosphorylation of S6K and 4E-BP1, downstream targets of mTOR, up to 48 h. The phosphorylation of S6, a downstream target of S6K, was correlated with the level of phospho-S6K. However, the level of phospho-eIF4E was elevated by LY294002, contrary to the reduction by the ERBB3 knockdown (Figure 6F,). LY294002 did not alter the phosphorylation of the MAPK pathways generally (Supplemental Figure 3B).

While the differential downstream pathways are inactivated, both the ERBB3 knockdown and LY294002 are able to inhibit cell proliferation efficiently. Thus, we examined the effects of co-treatment with siERBB3 and LY294002 on cells. Co-treatment with siERBB3 and LY294002 caused a more severe anti-proliferation than either treatment alone (Supplemental Figure 5A). Cell cycle analysis displayed a substantial increase in the G1 phase by co-treatment (Supplemental Figure 5B). However, there was no increase in the sub-G1 (Supplemental Figure 5B) or Parp1 cleavage (Supplemental Figure 5C), indicating that the additive inhibition of cellular proliferation by co-treatment is mainly due to increases in the G1 arrest without changes in apoptosis.

### ERBB3 knockdown induces anti-proliferation in the various human colon cancer cell lines

**Figure 7:**
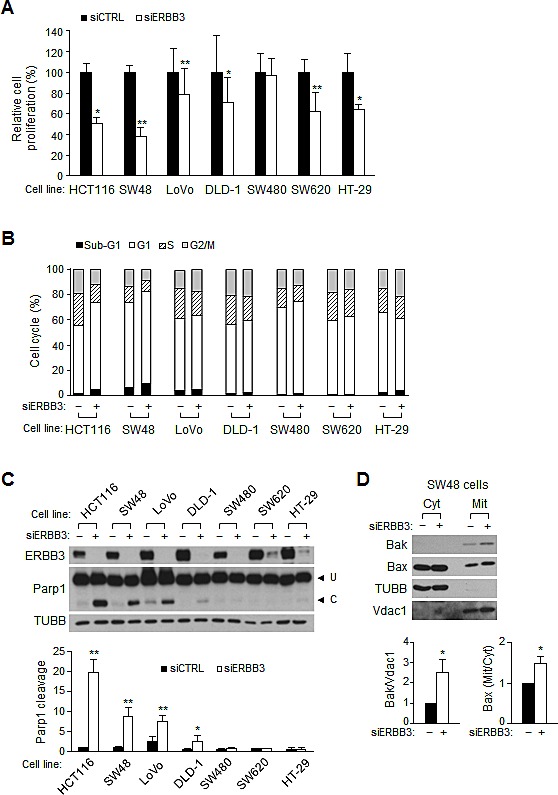
The ERBB3 knockdown-induced apoptosis and cell cycle arrest in human colon cancer cells Cells were treated with transfection reagent only (–) or siERBB3 (+) for 72 h. (A) Viable cells were counted after siRNA treatment and relative cell proliferation was compared to that of siCTRL in each cell line. (B) Cell cycle distribution was analyzed with FACS after siRNA treatment. (C) Western blotting was performed using an equal amount of protein extract (top), and the apoptotic index was determined by the ratio of cleaved to uncleaved Parp1 (bottom). (D) Protein extracts in the cytosolic or mitochondrial fraction were analyzed by western blotting in SW48 cells. The relative band intensities of the mitochondrial Bak to Vdac1 and of the mitochondrial Bax to cytosolic Bax were normalized to those of siCTRL. siERBB3 group was statistically compared to siCTRL group at each point.

A spectrum of mutations found in human colon cancer cells (http://cancer.sanger.ac.uk/cancergenome/projects/cell_lines/) may alter ERBB3 downstream signaling. To investigate whether ERBB3 targeting is still effective across such diverse mutations, we analyzed the effect of ERBB3 knockdown on several colon cancer cells with elevated ERBB3. ERBB3 knockdown caused the retardation of cell proliferation in the majority of cell lines, except for SW480 (Figure 7A). FACS analysis showed that ERBB3 knockdown led to G1 arrest in SW48, SW480 and SW620 cells like as HCT116 cells or G2/M arrest in LoVo, DLD-1 and HT-29 cells (Figure 7B), consistent with the previous report [25]. Parp1 cleavage also occurred in half of the cell lines examined (Figure 7C). The ERBB3 knockdown-induced upregulation of Bak and mitochondrial Bax translocation were also detected in the SW48 cells (Figure 7D) harboring wild-type PIK3CA. Overall, the ERBB3 knockdown resulted in G1 or G2/M arrest and concomitant apoptosis in some cell lines, indicating that ERBB3-targeted therapy may be effective, despite the varying mutations.

## DISCUSSION

Targeting the ERBB family inhibits tumor growth by suppressing downstream pathways, including the MAPK and AKT pathways, which are associated with cell proliferation and survival [49]. However, mutations downstream of ERBB that activate either pathway limit the clinical outcomes of ERBB-targeted cancer therapy [50]. In colorectal cancers, *KRAS* mutations are known to determine the clinical efficacy [51] and acquired resistance to EGFR targeting [52, 53]. In addition, either PI3KCA mutations or the loss of *PTEN* expression affects the resistance of cells to EGFR-targeted mAb [40]. Tumorigenic HCT116 cell growth is mainly due to heterozygous substitution mutations in KRAS G13D [42] and PIK3CA H1047R [43], which confer resistance to EGFR [39, 40] or ERBB2 [41] targeting by the constitutive activation of the MAPK and AKT pathways [43, 44], respectively. Indeed, the inhibition of both pathways efficiently induces anti-tumorigenesis [44, 54, 55], including elevated apoptosis [44, 55]. Contrary to resistance to EGFR or ERBB2 targeting in HCT116 cells, the ERBB3 knockdown alone sufficiently causes an attenuation of cell growth by inhibiting proliferation and elevating apoptosis, suggesting the presence of ERBB3-specific signal pathways that affect HCT116 cell growth.

The apoptosis induced by EGFR or ERBB2 targeting is mediated primarily by perturbing the BH3-only proteins or, alternatively, by regulating the expression of the anti-apoptotic Bcl-2 family, depending on the cell types. Previous reports have shown that Bad in breast cancer cells [56] or Bim in NSCLC [32-34] can integrate the MAPK and AKT signals for cell survival and death in the EGFR blockade. The significant induction of apoptosis by ERBB3 inhibition in HCT116 cells is unexpected without the alteration of two major downstream pathways. Certainly, our results showed that a majority of BH3-only proteins controlled by the MAPK or AKT pathway were not directly correlated with the ERBB3 knockdown-induced apoptosis. While increases in the amount of mitochondrial Bak and the Bax translocation, only observed in ERBB3 knockdowns but not in the LY294002 treatment, were crucial to induce apoptosis by ERBB3 inhibition. The activation of the pro-apoptotic Bcl-2 family, Bak and Bax, leads to apoptosis by forming channels to release cytochrome C and the subsequent activation of the caspase pathways [57]. Similar to the resistance of Bax^−/−^Bak^−/−^ DKO cells to various apoptotic stimuli [58], these cells are also resistant to ERBB3 knockdown-induced apoptosis. The even greater rate of apoptosis in Bak^−/−^ than in wild-type cells suggests that Bak is not needed for ERBB3 knockdown-induced apoptosis when Bax is present, consistent with the previous findings of predominant Bax-dependent apoptosis by various stimuli [58]. However, the moderate apoptosis in Bax^−/−^ cells indicates that while Bak can be activated independent of Bax, the latter is also required for the full induction of apoptosis. The significance of Bak activation is further confirmed here in its co-treatment with ERBB3 siRNA and LY294002, where less apoptosis correlates with less of an increase in Bak.

Because approximately 40-50% of colorectal cancers harbor *TP53* mutations (http://p53.free.fr/Database/p53_cancer/p53_Colon.html), ERBB-targeted therapies should be considered under the *TP53* status. p53 is involved in apoptosis by regulating the transcription of *BAX*, *PUMA* and *NOXA* [59], as well as the Bax translocation [60]. The absence of Bax translocation seems to be the primary reason for the lower rate of apoptosis against ERBB3 inhibition in p53^−/−^ cells. The significant amount of basal Bak in p53^−/−^ cells does not cause an elevated rate of apoptosis, which indicates that ERBB3 knockdown-induced apoptosis requires Bak activation beyond *BAK1* transcription. Both p53 and p73 play a major role in DNA damage-induced *BAK1* transcription in HCT116 cells [61], however, a slight *BAK1* mRNA increase without p53 and p73, similar to that in the ERBB3 knockdown, suggests the presence of p53- and p73-independent transcription regulation. The observation that basal or ERBB3 knockdown-induced *BAK1* mRNA levels are similar in wild-type, p53^+/+^ and p53^−/−^ cells also indicates the p53-independent regulation of *BAK1* expression. Although p53 mediates cell cycle arrest, the observation here that p53 doesn't affect ERBB3 knockdown-induced G1 arrest in p53^−/−^ cells indicates that cell cycle arrest by ERBB3 inhibition is independent of p53 in a wide range of colon cancer cells. However, HCT116, SW48 and LoVo cells which harboring wild-type *TP53* show a more apoptosis, supporting that p53 plays a role in apoptosis by ERBB3 inhibition.

Similar to other colon cancer cells [62], we report here that LY294002 exerts anti-tumorigenic effects on HCT116 cells by inhibiting their cell proliferation. The concentrations in our experiments (20 μM) did not cause apoptosis, despite the efficient downregulation of PI3K/AKT/mTOR pathway followed by the reduction of phospho-S6K and phospho-4E-BP1, both of which are controlled by mTOR directly [63]. The cell cycle arrest without apoptosis in HCT116 cells by another PI3K inhibitor [64], together with our report, indicates that the anti-tumorigenic effect of PI3K inhibition may result from the reduced cell proliferation, but not survival. The 4E-BPs inhibit cap-dependent translation by sequestering the eIF4E, and the phosphorylation of 4E-BPs dissociates eIF4E to make the eIF4F complex necessary for cap-dependent translation [47]. A 4E-BP1 is known to play a key role in integrating the AKT and MAPK pathways in HCT116 cells, as the inhibition of both pathways efficiently downregulates phospho-4E-BP1 [43, 44]. Moreover, the forced expression of a mutant 4E-BP1 with a defect in the phosphorylation sites was shown to suppress xenograft tumor growths [44]. To our surprise, the ERBB3 knockdown results in rapid and consistent decreases in the phospho-4E-BP1 slightly, without any significant changes of phospho-mTOR and phospho-S6K, indicating that the PI3K/AKT/mTOR-independent pathway that regulates phosphorylation of 4E-BP1. This attenuation of phospho-4E-BP1 and the downregulation of eIF4E level together seem to reduce the availability of free eIF4E and have been sufficient to downregulate the translation of Bcl-xL, Mcl1, Birc5 and Cyclin D1, which are sensitive to cap-dependent translation initiation [48] and subsequent attenuation of proliferation.

Overall, the ERBB3 blockade resulted in anti-tumorigenic effects without altering the PI3K/AKT/mTOR pathways in HCT116 cells by inhibiting cell proliferation or elevating Bak- and Bax-dependent apoptosis. Moreover, ERBB3 inhibition can provide significant anti-tumorigenic actions against colon cancer cells, despite the *KRAS*, PI3KCA and *TP53* mutations. Similar to the finding of TBK1 as a potent target for HER2-positive breast cancer [65], an identification of molecular targets regulated by ERBB3 signal pathways will be beneficial to design a new regime for ERBB3-targeted therapy or a combination therapy for treating colorectal cancers.

## METHODS

### Cell culture

Colon cancer cells were obtained from ATCC (Manassas, VA, USA) or KCLB (Seoul, Korea). DLD-1, LoVo, SW48, SW480 and SW620 cells were maintained in RPMI1640 medium supplemented with 10% fetal bovine serum (FBS) at 37°C in a humidified atmosphere of 5% CO_2_. Wild-type, p53^+/+^, p53^−/−^, Bax^−/−^, Bak^−/−^, Bax^−/−^Bak^−/−^ DKO HCT116 [58, 66, 67] and HT-29 cells were maintained in McCoy's 5A medium supplemented with 10% FBS. Both floating and adherent cells were harvested for the cell proliferation assay or FACS analysis, whereas adherent cells were washed twice with cold PBS and immediately stored at −80°C for RNA or protein extraction.

### Transfection of cells with siRNA or DNA

To adjust cell confluency at the time of harvesting, 6×10^5^ (treatment for 3, 6 or 12 h), 4×10^5^ (for 24 h), 2×10^5^ (for 48 h) and 1×10^5^ (for 72 h) cells were seeded 18 h before siRNA treatment, respectively. siRNA duplex oligonucleotides (Life Technologies, Carlsbad, CA, USA) were delivered into cells as described previously [29]. The sense sequences for ERBB3 siRNA (siERBB3) are, 5'-GGCCAUGAAUGAAUUCUCUACUCUA-3', 5'-GGAAGUUUGCCAUCUUCGUCAUGUU-3', and 5'-AGGACCAGAGCUUCAAGACUGUUUA-3'. For ERBB3 knockdown, equimolar ratio of three siRNA was mixed and scrambled duplex with high GC content (Life Technologies) was used for control siRNA (siCTRL). Bak siRNA (siBak) duplex (Genolution Pharmaceuticals, Seoul, Korea) is 21 bp including 3' UU-overhangs. The sequence of the siBak oligonucleotides is 5'-CCGACGCUAUGACUCAGAG -3'. The siCTRL for Bak knockdown is 5'-CCUCGUGCCGUUCCAUCAGGUAG -3'. For the knockdown of Bak and ERBB3 together, siBak transfection was followed by siERBB3 transfection 24 h later. *Erbb3* cDNA expression vector was constructed by subcloning of mouse full length *Erbb3* cDNA (Thermo Scientific, Pittsburgh, PA, USA) into pcDNA3.1. HCT116 cells at 70% confluency were transfected with 2.5 μg of cDNA expression vector or pcDNA3.1 vector using LT1 reagent (Mirus Bio, Madison, WI, USA) and then the cells (2×10^5^) were seeded 24 h after DNA transfection followed by further transfection with 5 nM of siRNA 24 h later.

### LY294002 treatment

Different number of cells, 6×10^5^ (treatment for 6 or 12 h), 4×10^5^ (for 24 h) and 2×10^5^ (for 48 h) were seeded 20 h before LY294002 treatment, respectively. LY294002 stock solution (10 mM, EMD Millipore, Darmstadt, Germany) was diluted with dimethyl sulfoxide (DMSO) and equal amount of solution was added (final DMSO concentration is 0.2%).

### Cell proliferation assay

Cell proliferation was quantified by counting the live cells after staining with trypan blue (Sigma-Aldrich, St. Louis, MO, USA).

### Cell cycle analysis with FACS

Cells (1 × 10^6^) were washed in PBS once and fixed in 70% ethanol for 30 min at −20°C. The fixed cells were washed once with PBS, resuspended in 1 ml PBS and treated with 0.1 mg/ml of RNase A (Sigma-Aldrich) for 10 min at room temperature. The cells were stained with 50 μg/ml of propidium iodide (EMD Millipore) for 10 min on ice and DNA content of cells was measured by flow cytometer (FACSCaliber, BD Bioscience, San Jose, CA, USA).

### Preparation of cell extracts and western blot analysis

Cell extracts were prepared by scraping the cells with 60-80 μl of lysis buffer (20 mM HEPES, pH 7.4, 150 mM NaCl, 10% glycerol, 1% Triton X-100, 1 mM PMSF, 10 μg/ml of leupeptin, 10 μg/ml of aprotinin, 1 mM Na_3_VO_4_, 1 mM NaF and 10 mM β-glycerophosphate) at 4°C and stored on ice for 10 min. Clearing the homogenates, protein quantification, SDS-PAGE, western blotting and quantification of blots were performed as described previously [29]. The antibodies against phospho-EGFR (LF-PA20127) were from Ab Frontier (Seoul, Korea); PDK1 (611070) from BD Biosciences; 4E-BP1 (9644), phospho-4E-BP1 (2855), Akt (9272), phospho-Akt (2965, 9271), Bad (9239), phospho-Bad (9296, 5286, 9297), Bak (3814), Bax (2772), Bcl-2 (2870), Bcl-xL (2764), Bid (2002), Bim (2819), Bok (4521), Cyclin D1 (2926), phospho-ErbB2 (2247), eIF4E (2067), phospho-eIF4E (9741), MAPK1/3 (4695), phospho-MAPK1/3 (9101), Mcl-1 (4572), RPS6 (2212), phospho-RPS6 (2211, 4858), RPS6K (9202), phospho-RPS6K (9204, 9208), p38 (9212), phospho-p38 (9211) Parp-1 (9542), phospho-PDK1 (3061), Puma (4976), SAPK/JNK (9258), phospho-SAPK/JNK (9251), mTOR (2983) and phospho-mTOR (2971, 5536) from Cell Signal Technology (Danvers, MA, USA); EGFR (06-129) and Noxa (114C307) from EMD Millipore; Cyclin E1 (M-20), ERBB2 (C-18), ERBB3 (C-17), c-Myc (C-19), p53 (DO-1), Birc5 (FL-142) and Vdac1 (N-18) from Santa Cruz Biotechnology Inc. (Dallas, TX, USA); β-actin (AC-15) and β-tubulin (D66) from Sigma-Aldrich, respectively.

### Quantitative real-time PCR (qRT-PCR)

Total RNA preparation, reverse transcription, real-time PCR and quantification of mRNA were performed as described previously [68] except using KAPA SYBR FAST qPCR kit (KAPA Biosystems, Wilmington, MA, USA) and *GAPDH* as an endogenous control. The sequence of genes for PCR is *BAK1,* 5'-tgcaacctagcagcaccatg-3' (forward) and 5'-actctcaaacaggctggtgg-3' (reverse); *BCL2L1,* 5'-cagcatatcagagctttgaacag-3' (forward) and 5'-gcattgttcccatagagttcc-3' (reverse); *BIRC5,* 5'-tgacgaccccatagaggaac-3' (forward) and 5'-ttctccgcagtttcctcaa-3' (reverse); *MCL1,* 5'-ggacgagttgtaccggcagt-3' (forward) and 5'-tttccgaagcatgccttgga-3' (reverse); *GAPDH,* 5'-ggaaggactcatgaccacagt-3' (forward) and 5'-cagtgagcttcccgttcag-3' (reverse).

### Preparation of cytosol and membrane/organelle fractions

Cytosol and membrane/organelle fractions were prepared using a ProteoExtract subcellular proteome fraction kit (EMD Millipore) according to manufacturer's protocol with minor modification. Briefly, 5 × 10^5^ cells (both floating and attached cells) were harvested, washed once with 0.5 ml of cold PBS and resuspended in extraction buffer I supplemented with protease inhibitors. After gentle tapping the tubes, the supernatants (cytosol fraction) were collected by centrifugation at 500 ×g for 10 min at 4°C. The pellets were resuspended in extraction buffer II supplemented with protease inhibitors and the supernatants (membrane/organelle fraction) were collected by centrifugation at 6000 ×g for 10 min at 4°C.

### Statistical analysis

Experimental groups were compared with one-tailed Student's *t*-test using Graphpad Prism program. All experiments were repeated three to four times, unless otherwise indicated and means ± S.D. are shown in the figures. Statistically significant differences are marked with *p<0.05, **p<0.01 and ***p<0.001, respectively.

## SUPPLEMENTARY FIGURES


