# Supplementary material for: ERBB3 knockdown induces cell cycle arrest and activation of Bak and Bax-dependent apoptosis in colon cancer cells
Source: Oncotarget. 2014 Jun 11;5(13):5138–52. doi: 10.18632/oncotarget.2094 (PMC4148128; doi:10.18632/oncotarget.2094)
Supplement: Supplementary file 1 [file oncotarget-05-5138-s001.pdf]

ERBB3 knockdown induces cell cycle arrest and activation of Bak and Bax-dependent apoptosis in colon cancer cells

Supplementary Material

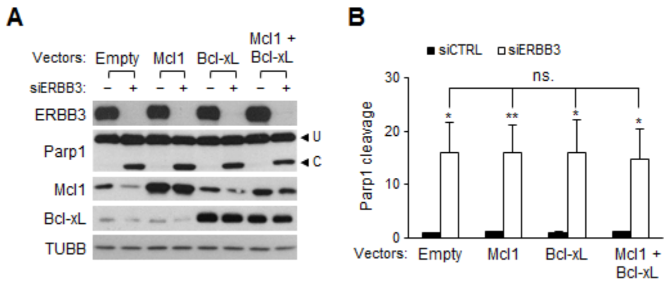

Supplemental Figure 1. No alterations in ERBB3 knockdown-induced apoptosis by the exogenous expression of Mcl1 or Bcl-xL in HCT116 cells. The Mcl1 cDNA expression vector was pcDNA3.1-MCL1 (Addgene plasmid 25375), and the Bcl-xL cDNA expression vector was pcDNA3.1-Bcl-xL constructed by cloning Bcl-xL cDNA fragments from the pSFFV-neo Bcl-xL (Addgene plasmid 8749) into pcDNA3.1. (A) Cells were analyzed with western blotting after cells were transiently transfected with cDNA expression vector or vector only (Empty), followed by siRNA treatment for 48 h. (B) The apoptotic index (Parp1 cleavage) was determined by the ratio of cleaved (C) to uncleaved Parp1 (U). siERBB3 group was statistically compared to siCTRL group at each point, unless otherwise indicated. ns., non-significant.

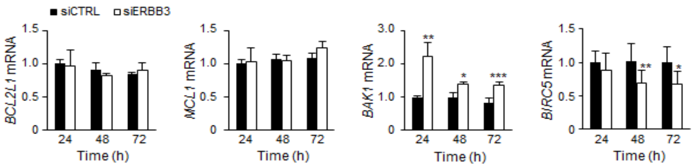

Supplemental Figure 2. ERBB3 knockdown-induced anti- or pro-apoptotic mRNA levels in HCT116 cells. The relative amounts of *BCL2L1*, *MCL1*, *BAK1* and *BIRC5* mRNA were analyzed by qRT-PCR analysis using RNA from cells harvested at the indicated time after siRNA treatment.

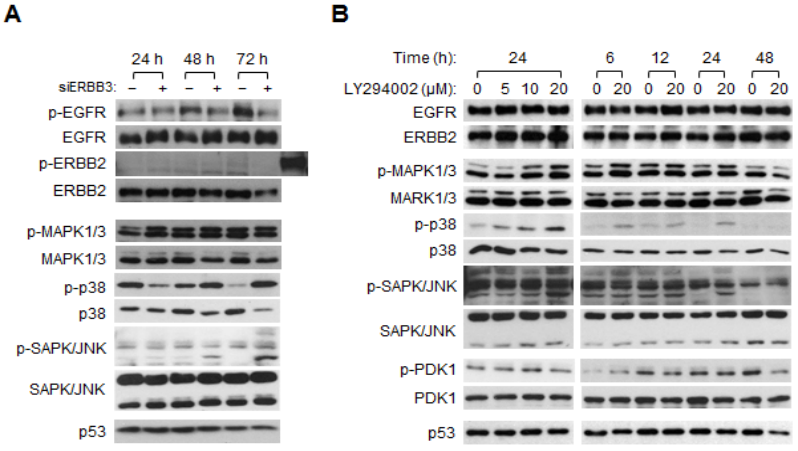

Supplemental Figure 3. Effect of siERBB3 or LY294002 on the EGFR family and MAPK signaling in HCT116 cells. (A) EGFR family and MAPK signal pathways were examined by western blotting daily after 5 nM of siCTRL (–) or siERBB3 (+) treatment. The extra lane in the p-ERBB2 is a positive control obtained from HT-29 cells. (B) Protein extracts were prepared either 24 h after treatment with a different concentration of LY294002 (left) or at different time points after treatment with 20 μM of LY294002 (right).

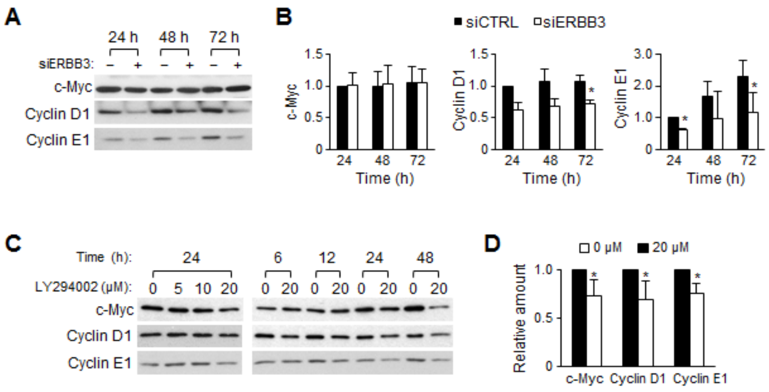

Supplemental Figure 4. The effects of siERBB3 or LY294002 on the cell cycle-related proteins in HCT116 cells. The amounts of c-Myc, Cyclin D1 and Cyclin E1 were measured by western blotting (A) daily after 5 nM of siCTRL (–) or siERBB3 (+) treatment and (B) the relative intensity of proteins compared to ACTB was normalized to that of siCTRL at 24 h. All experiments were repeated two times and means ± S.D. are shown. (C) The cell extracts were analyzed with western blotting 24 h after treatment with different concentrations of LY294002 (left) or at different time points after treatment with 20 μM of LY294002 (right), respectively. (D) The relative intensity of proteins (24 h after treatment with LY294002) compared to ACTB was normalized to that of DMSO-treated group at 24 h.

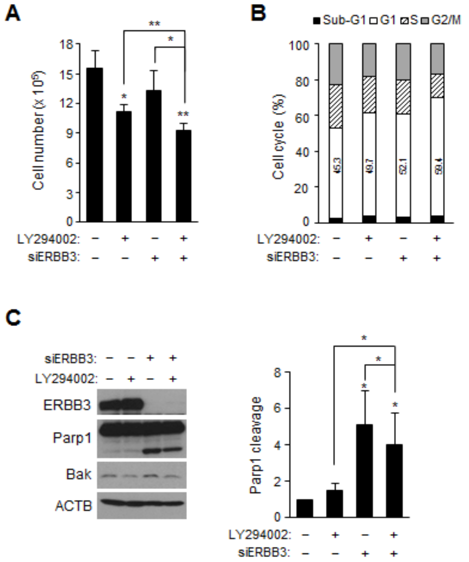

Supplemental Figure 5. Effect of co-treatment with siERBB3 and LY294002 on cell proliferation, cell cycles and apoptosis in HCT116 cells. Cells were transfected with 2 nM of siERBB3 (+) or siCTRL (–), followed by treatment with 10 μM of LY294002 (+) or DMSO (–) 24 h after siRNA treatment. (A) Viable cells, (B) cell cycle distribution and (C) western blots were analyzed 24 h after LY294002 treatment, respectively. Numbers in open box in B indicate a percent of G1 populations.
